# *Ke*tamin*e*/*p*ro*p*ofol *a*dmixture (ketofol) at induction in the *c*ritically ill against *e*tomidate (KEEP PACE trial): study protocol for a randomized controlled trial

**DOI:** 10.1186/s13063-015-0687-0

**Published:** 2015-04-21

**Authors:** Nathan J Smischney, Sumedh S Hoskote, Alice Gallo de Moraes, Carlos J Racedo Africano, Perliveh M Carrera, Rudy Tedja, Jasleen K Pannu, Elizabeth C Hassebroek, Dereddi Raja S Reddy, Richard F Hinds, Lokendra Thakur

**Affiliations:** Department of Anesthesiology, Mayo Clinic, 200 First Street SW, Rochester, MN 55905 USA; Department of Respiratory Care, Mayo Clinic, 200 First Street SW, Rochester, MN 55905 USA; Department of Medicine, Division of Pulmonary and Critical Care Medicine, Mayo Clinic, 200 First Street SW, Rochester, MN 55905 USA; Department of Anesthesiology, Division of Critical Care Medicine, Mayo Clinic, 200 First Street SW, Rochester, MN 55905 USA; Department of Critical Care Medicine, Geisinger Medical Center, 100 North Academy Avenue, Danville, PA 17822 USA

**Keywords:** Ketamine, propofol, drug combinations, etomidate, intubation, endotracheal, hemodynamics, adrenal insufficiency, randomized controlled trial, clinical research protocol, intensive care

## Abstract

**Background:**

Endotracheal intubation (ETI) is commonly performed as a life-saving procedure in the intensive care unit (ICU). It is often associated with significant hemodynamic perturbations and can severely impact the outcome of ICU patients. Etomidate is often chosen by many critical care providers for the patients who are hypotensive because of its superior hemodynamic profile compared to other induction medications. However, recent evidence has raised concerns about the increased incidence of adrenal insufficiency and mortality associated with etomidate use. A combination of ketamine and propofol (known as ketofol) has been studied in various settings as an alternative induction agent. In recent years, studies have shown that this combination may provide adequate sedation while maintaining hemodynamic stability, based on the balancing of the hemodynamic effects of these two individual agents. We hypothesized that ketofol may offer a valuable alternative to etomidate in critically ill patients with or without hemodynamic instability.

**Methods/design:**

A randomized controlled parallel-group clinical trial of adult critically ill patients admitted to either a medical or surgical ICU at Mayo Clinic in Rochester, MN will be conducted. As part of planned emergency research, informed consent will be waived after appropriate community consultation and notification. Patients undergoing urgent or emergent ETI will receive either etomidate or a 1:1 admixture of ketamine and propofol (ketofol). The primary outcome will be hemodynamic instability during the first 15 minutes following drug administration. Secondary outcomes will include ICU length of stay, mortality, adrenal function, ventilator-free days and vasoactive medication use, among others. The planned sample size is 160 total patients.

**Discussion:**

The overall goal of this trial is to assess the hemodynamic consequences of a ketamine-propofol combination used in critically ill patients undergoing urgent or emergent ETI compared to etomidate, a medication with an established hemodynamic profile. The trial will address a crucial gap in the literature regarding the optimal induction agent for ETI in patients that may have potential or established hemodynamic instability. Greater experience with planned emergency research will, hopefully, pave the way for future prospective randomized clinical trials in the critically ill population.

**Trial registration:**

Clinicaltrials.gov: NCT02105415. 31 March 2014.

**Electronic supplementary material:**

The online version of this article (doi:10.1186/s13063-015-0687-0) contains supplementary material, which is available to authorized users.

## Background

Endotracheal intubation (ETI) is a commonly-performed intervention in the intensive care unit (ICU). Critically ill patients are often hemodynamically unstable and have poor physiologic reserve, in contrast to patients undergoing ETI in an operating room [1]. Most of the medications used for sedation prior to ETI may precipitate or worsen hemodynamic instability [2]. In a prospective cohort study of 136 patients at an academic medical center, the rate of hemodynamic instability (systolic blood pressure <70 mmHg) occurred in about 10% of ETIs and was not influenced by the operator’s level of expertise [1]. Performing a randomized controlled trial in patients undergoing an urgent or emergent procedure poses several unique challenges, foremost of which is the issue of obtaining informed consent. Discussions with patients requiring ETI may be compromised by the patient’s mental status and may therefore be invalid. Furthermore, even if the patient is competent to make medical decisions, or the patient’s legally authorized representative is available, explaining the various aspects of risks and benefits, providing opportunity for questions, and allowing time for careful reflection may delay a vital and life-saving procedure. Therefore, a waiver of informed consent is essential in order to conduct such a study under planned emergency research.

As in the case of any clinical trial, clinical equipoise must exist in order to justify an experimental study on patients. The choice of the ideal sedative medication/combination for facilitating ETI remains unclear. Etomidate has proven to be efficacious in maintaining stable hemodynamics [3]. However, etomidate is an immediate and potent inhibitor of adrenal cortisol secretion [4] and has been associated with adrenal suppression and increased mortality in critically ill patients [4-10]. According to a recent meta-analysis, use of etomidate prior to ETI in the ICU was associated with a significantly increased risk of adrenal insufficiency (relative risk 1.64) and mortality (relative risk 1.19) compared to patients who did not receive etomidate prior to ETI [4].

Propofol is rapid in onset and has a short duration of action. In ICU patients, the most significant complication of propofol use is hemodynamic instability, which is secondary to reduction in systemic vascular resistance and myocardial depression [11-13]. This complication may be exaggerated in patients that have pre-existing hemodynamic compromise, by way of hypovolemia, sepsis, or cardiomyopathy. While this agent may be acceptable for hemodynamically stable patients who undergo ETI for elective purposes, it may be suboptimal if used alone in critically ill patients due to the risk of lowering mean arterial pressure (MAP).

Ketamine as a sole sedative has some concerns in the ICU for routine use. One head-to-head study comparing ketamine to etomidate in critically ill patients showed some benefits for ketamine; however, ketamine administration was done by emergency medicine physicians prior to ICU admission [14]. In addition, this study did involve waiver of consent due to the emergent nature of the intervention and the nonfeasible alternative of obtaining informed consent [14]. Ketamine use as a sole agent may have undesired sympathomimetic cardiovascular adverse effects, including elevations in blood pressure and heart rate, and arrhythmias [15-18]. These elevations in blood pressure and heart rate can have a detrimental effect on patients with underlying heart disease [19]. Additionally, ketamine may make intubation attempts difficult due to the increased muscular tone at certain dosage levels [20]. Evidence suggests that difficult intubations lead to increased mortality in critically ill patients from repeated airway manipulations [21,22]. Finally, emergence phenomenon or delirium is one of the most common and well-studied adverse effects after ketamine administration [23]. Several groups have evaluated medications such as dexmedetomidine, benzodiazepines or propofol when used as adjuncts or premedications with ketamine to attenuate emergence delirium [24-26].

Benzodiazepines are not ideal as evidence demonstrates an increased risk of both delirium and mortality with their use [27,28]. Barbiturates are also unsatisfactory as they may also lead to an undesirable decrease in mean arterial pressure, secondary to a decreased systemic vascular resistance and decreased cardiac output.

In recent years, an admixture of propofol and ketamine (known as “ketofol”) has been studied in a variety of clinical settings, including sedation for ETI, gastrointestinal endoscopy, bronchoscopy, and emergency department procedures [12,29-33]. Since the adverse effects of ketamine are dose related, a reduction in dose by using ketamine in combination with propofol may improve these adverse outcomes. Therefore, the use of smaller doses is likely to be beneficial as evidenced by recent case reports of septic shock patients who suffered cardiac arrest after receiving 2 mg/kg of ketamine [34]. The combination of ketamine and propofol may potentially balance each other’s hemodynamic adverse effects and, therefore, offer a safer alternative of sedation prior to ETI.

In summary, etomidate and ketamine-propofol combination appear to have the best efficacy and safety profile amongst anesthetic agents used for sedation prior to ETI. There is sufficient equipoise regarding the choice of the best induction agent or combination among these agents, for critically ill patients undergoing ETI. This forms the rationale for testing a combination of ketamine and propofol against etomidate in this patient population.

The current trial, *Ke*tamin*e*/*P*ro*p*ofol *A*dmixture “Ketofol” at Induction in the *C*ritically Ill Against *E*tomidate (KEEP PACE trial) has been designed with the primary aim of assessing the hemodynamic consequences of “ketofol” compared to etomidate in critically ill patients undergoing urgent/emergent ETI.

### Hypotheses

- Primary hypothesis: The decrease in MAP for the “ketofol” arm will be less than the etomidate arm within 15 minutes post-drug administration in the population of critically ill patients needing urgent and/or emergent endotracheal intubation.
- Secondary hypothesis 1: The 28-day mortality among patients in the “ketofol” arm will be lower compared to the 28-day mortality in the etomidate arm.
- Secondary hypothesis 2: The use of vasoactive medications to restore the blood pressure post-drug administration will be reduced in the “ketofol” group compared to the etomidate group.
- Secondary hypothesis 3: The “ketofol” group will have a decreased incidence of adrenal insufficiency compared to the etomidate group, defined as a pre-cosyntropin cortisol of less than 10 mcg/dL or a post-cosyntropin cortisol increase by less than 9 mcg/dL after 250 mcg of cosyntropin.

## Methods/design

The current study protocol was approved by the Mayo Clinic Institutional Review Board for planned emergent use research. Institutional Review Board # 13–000506.

### Design and setting

This is an institutional review board (IRB)-approved randomized controlled, parallel-group, clinical trial of adult critically ill patients, with stratification and block randomization to “ketofol” and etomidate groups. The patients will be recruited from medical and surgical ICUs at Saint Marys Campus of Mayo Clinic Hospital in Rochester, MN. This hospital campus is a 1,157-bed facility with 55 operating rooms and approximately 200 total ICU beds. The medical ICU has 24 beds and the medical decision-making team consists of internists with pulmonary and critical care medicine training, critical care fellows, internal medicine residents, physician assistants and nurse practitioners. The surgical ICU has 20 beds and the medical decision-making team consists of anesthesiologists with critical care training, critical care fellows, anesthesiology residents, physician assistants and nurse practitioners. In the medical ICU, the critical care team makes all management decisions for the patient; while in the surgical ICU, critical medical decisions are usually taken after discussion between the surgical and critical care services. The above team members staff the ICU 24 hours a day and 7 days a week. All procedures in the ICU, including ETI, are performed by fellows or residents under direct supervision by attending critical care physicians. A bedside nurse and a respiratory therapist provide assistance with all ETIs in the ICU.

### Patient selection

Adult patients (age ≥18 years old) about to undergo ETI in the ICU will be evaluated for inclusion in the study. The intensive care attending physician must agree to follow the study plan and drug randomization for a patient to be included. Patients meeting the following criteria will be excluded: (i) ETI being performed in the setting of an elective procedure; (ii) known intracranial pathology such as acute intracranial hemorrhage or intracranial mass or acquired head injury; (iii) known chronic opiate dependence as defined by those patients on methadone, buprenorphine, buprenorphine-naloxone or naltrexone as an outpatient; (iv) continuous sedative intravenous infusions (propofol, midazolam, lorazepam, fentanyl or dexmedetomidine) for the past 24 hours; (v) known severe psychiatric illness, defined as bipolar disorder and/or schizophrenia; (vi) known egg allergy; (vii) known contraindications to administration of fentanyl, midazolam, ketamine, propofol or etomidate; (viii) intubation performed during true emergent situations, such as cardiac arrest, where standard practice is not to use induction drugs; (ix) body weight ≥140 kg or ≤30 kg, or no body weight documented in the medical record; or (x) prior participation in the current study. Additionally, special exclusion criteria will apply for women of childbearing age (defined as age 18 to 50 years old). Women in this age group who do not have a known documented negative pregnancy test at our institution, or who do not have a known surgical procedure preventing pregnancy (for example, tubal ligation or hysterectomy) will be excluded. If the pregnancy test result is unknown or whether the patient has undergone a surgical procedure preventing pregnancy is uncertain, the patient will not be included in the trial. The rationale for this strategy is to minimize as much as possible the time needed to obtain the necessary information in order to reduce potential harm to patients given the time-sensitive nature of ETI. All exclusion criteria will be posted on the outside of the randomization envelopes as a checklist to serve as an additional reminder to the care team before proceeding with inclusion for the study.

### Informed consent

In most circumstances, for patients requiring ETI in the ICU, the severity of the patient’s medical condition makes an informed consent discussion extremely challenging, or even invalid. Under the United States Food and Drug Administration (FDA) regulations, research in patients during medical emergencies is exempt from informed consent requirements provided certain conditions are met. The FDA defines emergency research as investigations that “involve human subjects who have a life-threatening medical condition that necessitates urgent intervention (for which available treatments are unproven or unsatisfactory), and who, because of their condition (for example, traumatic brain injury), cannot provide informed consent” [35]. Additionally, the FDA recommends that the research “must have the prospect of direct benefit to the patient and must involve an investigational product that, to be effective, must be administered before informed consent from the subject or the subject’s legally authorized representative (LAR) can be obtained and in which there is no reasonable way to identify prospectively individuals likely to become eligible for participation” [35]. The KEEP PACE trial meets these definitions to qualify as a planned emergency research study. In keeping with FDA recommendations [35], we conducted community consultations, by way of focus groups, to gather representatives from the community in which the investigation was planned, in order to disclose all aspects of the research protocol. The information from the focus groups was then used to create public notification strategies. Of note, the KEEP PACE Trial is under an Investigational New Drug (IND) protocol as studies conducted under planned emergency research require an IND/Investigational Device Exemption (IDE) from the FDA.

Community consultation and public disclosure were achieved through the following methods:

1. Focus groups: Four group sessions were conducted. One of these groups consisted exclusively of individuals who seek medical care at Olmsted Medical Center, which is the only other medical center serving residents of Olmsted County apart from Mayo Clinic. The rationale behind this focus group was to include community members that may potentially harbor reservations against receiving care at a large, academic medical center where medical research is carried out. Overall, the focus group members expressed support of the study and a willingness to participate in it themselves. They agreed that obtaining informed consent would be impractical. They expressed the need for a mechanism by which patients, after recovering from acute illness, would be given an option of withdrawing their data from the study. They agreed that developing a community-wide opt-out registry drawn out prior to starting the trial would not be necessary or helpful. Members suggested that public disclosure be carried out using local newspapers, newsletters, radio, and television, including ethnic radio and television programming.
2. Public disclosure plan: Disclosure will involve a summary of all key aspects of the study, including the nature of planned emergency research, study protocol, medications, patient participation, waiver of informed consent, and links to information about the study and the investigators. Throughout the duration of the study, notices of the study will be published in the Post-Bulletin (the largest daily newspaper in Southeastern Minnesota) approximately every 2 weeks and in local circulars with a different target audience (for example, locally circulated publications, and local radio shows (Mayo Medical Edge Weekend on KROC-AM). The principal investigator may also make appearances on public access television and local television shows to discuss the study, should such opportunities become available. A video of the principal investigator explaining the study will play on one of Mayo Clinic’s internal television channels, and will be shown in the patient rooms and family waiting areas in the two ICUs included for this study. This video has already been posted on the Mayo Clinic YouTube channel for public viewing [36]. Detailed information about the trial for the general public has also been posted on Mayo Clinic’s website [37]. Copies of the newspaper notices and notices for patients and relatives posted within the hospital are contained in Additional file 1. Once the trial begins, there will be a minimum of four meetings with members of the public to provide information and answer questions; and these dates will be communicated to the public via notices in local newspapers.
3. Physician involvement: A prerequisite for patient enrollment in the trial is agreement from the treating intensivist. The principal investigator presented the rationale and details of the protocol at several educational activities intended for intensivists, such as critical care grand rounds as well as lectures for fellowship trainees, and these talks were made available on the intranet for future reference by critical care providers. A poster with trial details was displayed in the ICUs and in the critical care division (see Additional file 2), and a slide show to explain trial details was included in the orientation materials sent out to residents and fellows before starting an ICU rotation. All incoming trainees at the start of the academic year also received a presentation about the trial during their on-boarding process.

### Randomization

A computer-generated stratified blocked randomization will be used, which will be generated by a statistician who is not involved in determination of patient eligibility, drug administration, or outcome assessment. Randomization will be performed using two stratification factors (ICU type: medical or surgical, and shock state: MAP ≥65 mmHg or MAP <65 mmHg). Within each stratum, randomization will be performed using blocks of size n = 4 to ensure that, after every 4th patient is randomized within a given stratum, there are an equal number of patients assigned to each treatment group. Randomization will be accomplished by using opaque envelopes in each ICU placed adjacent to the drug-dispensing machine (Pyxis MedStation™ system, CareFusion Corporation, San Diego, CA). The randomization envelopes will contain a card stating the drug to which the patient was randomized.

### Medications, doses and preparation

The key study drugs will be the following: ketamine (ketamine hydrochloride, USP; 20 ml vial; 10 mg/ml; manufactured by either JHP Pharmaceuticals, LLC, Parsippany, NJ or Mylan Inc., Canonsburg, PA), Diprivan™ (propofol injectable emulsion, USP; 20 ml vial, 10 mg/ml; manufactured by APP Pharmaceuticals, LLC; a division of Fresenius Kabi USA, LLC, Lake Zurich, IL), and Amidate™ (etomidate injection, USP; 10 ml vial; 2 mg/ml; manufactured by Hospira Inc., Lake Forest, IL). All medications will be provided through the drug dispensing machine in each ICU (Pyxis MedStation™ system, CareFusion Corporation, San Diego, CA). When ketamine and propofol are mixed in a 1:1 ratio, the resulting mixture will have a concentration of 5 mg/ml of ketamine and 5 mg/ml of propofol.

For patients randomized to receive “ketofol”, the assigned bedside nurse will draw one 20-ml vial each of propofol and ketamine, in an aseptic fashion, into a 60 ml syringe. The dose will be calculated as 0.5 mg/kg of ketamine and 0.5 mg/kg of propofol based on actual body weight, rounded up to the nearest weight increment. The weight-based dosing chart will be available within the randomization envelope (see Additional file 3). The 60-ml syringes used to deliver the study medications will contain the appropriate IND labeling and study subject identification, contained within the opaque envelope. The initial dose will be considered the “induction dose” and a second dose of equivalent strength (0.5 mg/kg of ketamine and propofol each, rounded up to the nearest weight increment) may be administered as a “rescue dose” if adequate sedation was not achieved with the induction dose. If the rescue dose also fails to provide adequate sedation, the remainder of the syringe may be used as per the discretion of the critical care clinician. We do not anticipate this will happen often, given the initial prescribed doses. However, the patient may not cross over to use etomidate after “ketofol” has been administered.

For patients randomized to receive etomidate, the assigned bedside nurse will draw two 10-ml vials of etomidate, in an aseptic fashion, into a 35-ml syringe. The dose will be calculated as 0.15 mg per kilogram of actual body weight, rounded up to the nearest weight increment. A weight-based dosing chart will be available within the randomization envelope (see Additional file 3). The 35 ml syringe used to deliver the study medication will contain the appropriate IND labeling and study subject identification number. The initial 0.15 mg/kg will be considered the induction dose and a second dose of 0.15 mg/kg will serve as a rescue dose if deemed clinically necessary. Should the clinical team need more than 0.3 mg/kg of etomidate to provide adequate sedation, they may use the remainder of the study syringe as determined by the critical care clinician. We do not anticipate this will happen often, given the initial prescribed doses. However, the patient may not cross over to use ketamine, propofol, or “ketofol” after etomidate has been administered.

“Ketofol” dosing calculations are based on prior work by Hui et al. [38], who showed that a 0.5 mg/kg dose of ketamine and propofol each produced anesthesia in roughly 50% of healthy patients who did not receive other sedatives and were not critically ill. We know that the critically ill are provided with additional sedatives for various reasons and that certain medications (for example, propofol) have increased potency in shock patients [39]. If the rescue dose is given in this study, the patient will have received a standard induction dose of ketamine. This is a standard induction dose for an elective surgical patient and not a critically ill patient. Furthermore, the patient will also have been given propofol. Conditions in which the critical care provider may need additional anesthetic agent during endotracheal intubation may include (but are not limited to) the following: (i) patient movement/purposeful response to the intervention; (ii) difficult intubation; or (iii) inadvertent underdosing. Therefore, the only decision made by the provider will center on whether they request the rescue dose and/or additional doses. The bedside nurse will be provided with a flowchart of tasks to be performed during the process of ETI. This flowchart will be contained within the randomization envelope. Again, both the flowchart and dosing chart will also be provided on the intubation equipment and Pyxis machine, outside the randomization envelope. The remaining study medications will be disposed through the Pyxis machine per usual practice.

### Intubation period

Patients will receive standard intensive care unit monitoring consisting of electrocardiogram analysis, pulse oximetry, and a noninvasive blood pressure cuff. The presence of invasive monitors such as an arterial line will be allowed. However, to standardize measurement techniques, all hemodynamic measurements will be based off the noninvasive devices. Once the decision to intubate the patient is made by the clinical team, both the unit Registered Respiratory Therapist (RRT) and the assigned bedside nurse will be informed as per usual practice. The unit RRT will notify the lead RRT to immediately be present at the patient’s bedside. The lead RRT or a delegate will serve as the recorder RRT and will collect the necessary information in real time and document it in the data collection form (see Additional file 4). Prior to ETI, the unit RRT will set the time intervals on the noninvasive blood pressure cuff monitoring and electronic medical record in the patient’s room to run every one minute until 15 minutes after successful intubation. The unit RRT will then assist with the intubation per usual practice. Monitoring will then be switched to 5-minute intervals for the remaining 45 minutes post-intubation for a total duration of 60 minutes post-intubation. Hemodynamics recorded one minute prior to induction will be considered as a baseline. At induction, the trial drug will be administered over 60 seconds along with fentanyl at 50 mcg. Nursing staff will document the volume of drug administered in the electronic medication record and the volume will be verified with the volume recorded by the recorder RRT for accurate study drug reconciliation. Neuromuscular blockade as deemed necessary by the critical care provider will be allowed. Midazolam as deemed necessary by the critical care provider will be allowed. Intubation times will be recorded by the recorder RRT. Intubation attempt is manipulation of the airway by any device inserted into the pharynx. Intubation time is the time when the airway is established by confirmatory end-tidal C02 monitoring. The time from study drug administration to intubation will not be standardized. After intubation, sedation is then to be maintained with the choice decided by the critical care provider. Additional narcotics will be allowed as necessary throughout the study period. If necessary, anti-cholinergics, vasoactive and steroid medications will be allowed as well. Hemodynamics (MAP, systolic blood pressure, diastolic blood pressure, and heart rate) will be recorded periodically, as detailed below. Time zero would be defined as the time of study drug administration. Emergence from anesthesia will not be controlled by the study protocol.

At our institution, all intubations adhere to our intubation checklist, which includes a discussion of alternative airway techniques for a possible difficult airway. The use of neuromuscular blockers was not strictly protocolized as some clinicians would be unwilling to participate in the current study and thus, the feasibility would be substantially affected. Also, some clinicians would be unwilling to participate in the trial as the use of neuromuscular blockers may be detrimental in a difficult airway (loss of spontaneous respirations). Furthermore, application of standardized procedures of sedation and intubation would simulate more of a scientific rather than a pragmatic protocol and also affect feasibility/enrollment.

### Study measures and assessments

- Hemodynamic Assessment: Noninvasive blood pressure measurements will be obtained from one minute prior to study drug administration to one hour after successful intubation. Even if the patient has an intra-arterial (invasive) blood pressure monitoring, only the noninvasive blood pressure will be collected for analysis. This will be done in order to standardize the measurement method across patients. For the first 15 minutes post-administration and until 15 minutes after successful intubation, measurements will be taken every 1 minute. Following this interval, the measurements will then be switched to intervals of 5 minutes until the time period of one hour has elapsed since successful intubation. Data will be obtained from the chart from the period of 1 hour prior to intubation and 1 hour after intubation.
- Clinical Assessment: General characteristics of the patient including demographics, hemodynamics (average/median of values 60 minutes prior to intubation), cardiovascular medications, and acute physiology and chronic health evaluation (APACHE) 3 score for the time period of 24 hours prior to study drug administration will be obtained. Final diagnosis will be recorded. Interventions including transfusions, total fluid volume, urine output, total amount of analgesic/sedative medications utilized, vasopressor/steroid administration, confusion assessment method for the intensive care unit (CAM-ICU) scores and mechanical ventilation parameters will be recorded during the study period of 24 hours pre- and post-drug administration. Provider satisfaction will be assessed in both groups through the use of surveys provided to the intubating physician after completion of successful intubation. The Cormack-Lehane grade and intubation difficulty scale will be recorded (see Additional file 4).
- Adrenal Assessment: A cosyntropin stimulation test will be performed at approximately the 4 and 24 hour mark by drawing serum cortisol levels approximately 1 hour before and 1 hour after 250 mcg cosyntropin. The test will be considered normal if the pre-cosyntropin cortisol is greater than 10 mcg/dL or if the post-cosyntropin cortisol rises by more than 9 mcg/dL [40]. This will be accomplished through the use of the Clinical Research Unit (CRU) and the Center for Clinical and Translational Science (CCaTS) immunology core.
- Quality Control Measures: The quality of randomization will be accounted for by the study coordinator and lead respiratory therapist. They will ensure an accurate record of those participants who received a particular intervention. We will utilize the REDCap (Research Electronic Data Capture) data management system (REDCap Software, version 4.13.17, Vanderbilt University, Nashville, TN). Weekly reports will be generated from REDCap to assess whether this has been completed correctly. Additionally, this system will include data entry forms that will be filled out by the study coordinator and other investigators, after receiving a page for a study subject, during the study, and then entered into the REDCap system at the end of the week. Performance reports will be generated weekly to ensure that any missing values or out-of-range entries are flagged and responded to appropriately. Periodic reports every month will be generated to assess progress of study and address any concerns. In addition, the unit critical care charge nurses will be contacted twice monthly to address any concerns that may have surfaced from the study until study completion. Furthermore, the physicians in training will be contacted each month on the first day of duty and informed of the study until study completion. The first ten patients will be assessed for errors within the devised system and any adjustments needed prior to further enrollment. Interim analysis will be conducted to ensure patient safety regarding mortality and serious adverse events to the patient. Regarding ketamine, emergence phenomenon is a concern and this will be assessed by recording (CAM-ICU) scores in the intensive care unit during the follow-up period. To ensure proper doses of medications given, tables will be provided to the bedside nurses within and outside the randomization envelopes with the correct dose of both drugs to be given according to the patient’s weight. Furthermore, a checklist containing the necessary steps (please refer to the intubation period) to be completed during the intubation period will be provided within and outside the randomization envelopes. The unit nursing team will be provided with online educational sessions through online training modules regarding the above process. Prior to study enrollment, there will be several unit staff meetings with both intensive care unit consultants and nurses. The times will be documented for training purposes and physicians who are not willing to participate in the study will be recorded.

### Data collection

Data collection will be carried out from several sources. A respiratory therapy supervisor (known as recorder RRT) will be given the responsibility of real-time data recording during the process of ETI. This data will include the patient’s study identification number, shock status (MAP ≥65 mmHg or MAP <65 mmHg), dose and timing of each medication administered during and up to 15 minutes after successful ETI, name and rank of the medical provider performing ETI, lot number and expiration date of the study drug vials (etomidate, ketamine or propofol) used during ETI, and whether the patient suffered a cardiac arrest during the ETI (see Additional file 4). After the ETI is completed, the provider performing the ETI will fill out a separate sheet to record the intubation difficulty scale, Cormack-Lehane grade and whether any procedures were planned within 60 minutes of the ETI (see Additional file 4). After the initial 1-minute intervals for noninvasive blood pressure monitoring 15 minutes post-intubation, blood pressure measurement will be switched to every 5 minutes until 60 minutes of recording post-intubation. This will subsequently be changed to every 15 minutes of recording which is the usual practice. Trained data abstractors will compile these two paper forms and enter them into a REDCap database. The remainder of the clinical data, as described above, will be collected using METRIC Data Mart, a validated and reliable database tool for recording essential data in ICU patients [41].

### Sample size calculation

The sample size for the current study was based on the assumption that the standard deviation for the change in MAP from baseline to 5 minutes post-induction is approximately 10 mmHg, and that the mean difference between groups will need to be at least 5 mmHg in order to be considered clinically relevant. Based on these assumptions, we determined that a sample size of N = 64 per group will provide statistical power (two-tailed, alpha = 0.05) of 80% to detect a clinically relevant difference (that is, difference of 5 mmHg) between groups. We designed the study to have adequate statistical power to detect a 5 mmHg difference between groups using a two-sided test. In addition to performing the hypothesis test, we will also calculate a 95% confidence interval for the difference between groups. If we do not find a statistically significant improvement, the bounds of the 95% confidence interval should allow us to make a conclusion regarding noninferiority. The effective sample size (that is, the number of subjects who have data for the primary outcome) required will be 128. Increasing the sample size to allow for 20% subject attrition (drop-out) will be undertaken given the critical nature in which the study will be conducted. Increasing the sample size to 160 would allow for 20% drop-out (that is, 80% of 160 = 128). Therefore, we will enroll a total of 160 research participants.

The primary endpoint for the current investigation is change in MAP from baseline to 5 minutes post-induction. For this endpoint, we consider a difference between groups of 5 mmHg to be clinically relevant and have designed the study to have adequate power to detect this magnitude of a difference. We chose this endpoint because induction medications are normally redistributed out of the central circulation after 5 minutes. Furthermore, the morbidity endpoint of hemodynamic instability, for example, post-intubation hypotension, was chosen rather than the mortality endpoint as critically ill patients who develop post-intubation hypotension of any duration have an associated increased mortality and length of stay. This was demonstrated recently by a retrospective study addressing the incidence of post-intubation hypotension and its possible association with in-hospital mortality. The authors defined post-intubation hypotension as a systolic blood pressure less than 90 mmHg occurring within 60 minutes of emergency intubation. Post-intubation hypotension occurred in over 20% of the 465 patients who underwent emergent intubations and was associated with significantly higher in-hospital mortality and longer intensive care length of stay [42,43]. Although we will report the frequency of hypotension for each group, we have not designed the study to provide adequate statistical power for this endpoint.

### Data analysis

Descriptive statistics will be used to summarize demographic information and other baseline characteristics. These summaries will be generated for the entire sample, as well as according to treatment group. Given the randomized study design, we will not perform formal hypothesis testing of baseline characteristics to assess the statistical significance of differences between treatment groups. For each analysis, model assumptions will be validated, with transformations or nonparametric methods utilized, as appropriate. In all cases, findings will be summarized using point-estimates and 95% confidence intervals; two-tailed *P* values ≤ .05 will be used to denote statistical significance.

#### Primary analysis

For the primary specific aim, the outcome of interest is change in MAP from baseline over the first 5 minutes following induction. Data will be recorded at baseline and every minute thereafter. The primary endpoint will be the change in MAP at 5 minutes post-drug administration. For the primary analysis, the change in MAP from baseline to 5 minutes post-induction will be compared between groups using analysis of covariance with shock state (MAP ≥65 versus MAP <65) and number of doses included as covariates. From this analysis, the estimated difference between treatment groups will be summarized using a point estimate and 95% confidence interval. Secondary analyses will be performed to compare MAP changes from baseline to 10 and 15 minutes as well as the average MAP area under the curve (AUC) over the first 15 minutes expressed as a change from baseline. We will assess the treatment-by-shock state interaction effect to assess whether treatment differences are dependent on this stratification factor. Additionally, we are particularly interested in the subgroup of patients on vasoactive medications and those who have received at least 30 ml/kg of fluid within three hours prior to intubation. Therefore, additional analyses will be performed to compare treatment groups within each of these subgroups.

The 5-minute mark was chosen as the primary outcome focuses on hemodynamics of induction medications. After 5 minutes, induction medications are typically redistributed out of the central circulation and into the periphery, thus, abating the hemodynamic effects of the indication agents. The morbidity endpoint of hemodynamic instability, for example, post-intubation hypotension was chosen rather than the mortality endpoint as critically ill patients who develop post-intubation hypotension of any duration have an associated increased mortality and length of stay [42,43].

#### Secondary analyses

The vital status (alive/dead) of each patient at 28 days following their randomization will be obtained through a review of medical charts. The percentage of patients who die within the first 28 days will be summarized for each treatment group and compared between treatment groups using Fisher’s exact test (**Secondary Aim 1**). To determine whether the “ketofol” is associated with a decreased use of vasoactive medications (**Secondary Aim 2**), the percentage of patients who receive vasoactive medications in the first hour following treatment administration will be compared between treatment groups using Fisher’s exact test. In order to assess whether the “ketofol” is associated with a decreased incidence of adrenal insufficiency (**Secondary Aim 3**), the percentage of patients who meet the criteria for adrenal insufficiency from either of the cosyntropin stimulation tests performed at 4 and 24 hours will be compared between groups using Fisher’s exact test.

#### Additional analyses

Although not specifically included in the study aims, a number of additional secondary endpoints will be evaluated. These endpoints include the number of mechanical ventilation-free days at day 28, intensive care unit-free days at day 28, vasoactive free-days until day 28, transfusions, *etcetera* These endpoints will be compared between treatment groups using appropriate two-sample methods (for example, Wilcoxon rank sum test, Fisher’s exact test).

### Protection of human subjects

- Consent: As discussed above, a waiver of informed consent will apply due to classification of this trial as planned emergency research. Patients will have the option of retracting their consent for participation once they are clinically stable to participate in an informed consent discussion. Patients choosing to retract consent will have their data excluded from the study.
- Data Protection: To ensure the protection of patient data, all study data is stored on an encrypted, restricted access server maintained behind Mayo Clinic’s firewall. Only study staff will have access to this information. In the event that paper documentation exists for research subjects, their research folders will be maintained in a locked cabinet in a building with restricted access. Only study staff will have access to the files. After data analysis, study records will be stored for a duration according to regulation and subsequently destroyed.
- Assessment of Safety Adverse Event Definitions:
  - Adverse Event (AE): An adverse event is any untoward medical occurrence associated with the use of a drug in humans, whether or not considered drug related.
  - Life-threatening Adverse Event or Life-threatening Suspected Adverse Reaction: An adverse event or suspected adverse reaction is considered “life-threatening” if, in the view of either the investigator or sponsor, its occurrence places the patient or subject at immediate risk of death. It does not include an adverse event or suspected adverse reaction that, had it occurred in a more severe form, might have caused death.
  - Serious Adverse Event (SAE) or Serious Adverse Reaction: An adverse event is defined “serious” if, in the view of either the investigator or sponsor, it results in any of the following outcomes: death, a life-threatening adverse event, inpatient hospitalization or prolongation of existing hospitalization, a persistent or significant incapacity or substantial disruption of the ability to conduct normal life functions, or a congenital anomaly/birth defect. Important medical events that may not result in death, be life-threatening, or require hospitalization may be considered serious when, based upon appropriate medical judgment, they may jeopardize the patient or subject and may require medical or surgical intervention to prevent one of the outcomes listed in this definition.
  - Suspected Adverse Reaction: A suspected adverse reaction is any adverse event for which there is a reasonable possibility that the drug caused the adverse event. For the purposes of IND safety reporting, “reasonable possibility” means there is evidence to suggest a causal relationship between the drug and the adverse event. Suspected adverse reaction implies a lesser degree of certainty about causality than adverse reaction, which means any adverse event caused by a drug.
  - Unexpected Adverse Event or Unexpected Suspected Adverse Reaction: An adverse event or suspected adverse reaction is considered “unexpected” if it is not listed in the investigator brochure or is not listed at the specificity or severity that has been observed; or, if an investigator brochure is not required or available, is not consistent with the risk information described in the general investigational plan or elsewhere in the current application, as amended.
- IND Safety Reports: The study team will notify the FDA and all participating investigators (that is, all investigators to whom the sponsor is providing drug under its INDs or under any investigator’s IND) in an IND safety report of potential serious risks, from clinical trials or any other source, as soon as possible, but in no case later than 15 calendar days after the sponsor determines that the information qualifies as “Serious and Unexpected Suspected Adverse Reaction”. Results of other studies suggesting significant risk to humans exposed to drugs used in this trial, any trials conducted in animals or in-vitro testing that suggests significant risk to humans exposed to the drug, or a clinically significant increased rate of occurrence of serious suspected adverse reactions will be reported. In each IND safety report, the sponsor must identify all IND safety reports previously submitted to FDA concerning a similar suspected adverse reaction, and must analyze the significance of the suspected adverse reaction in light of previous, similar reports or any other relevant information. Adverse events possibly related to etomidate or “ketofol” administration include but are not limited to tachycardia/bradycardia, hypotension/hypertension, nausea/vomiting, hallucinations, disorientation, anxiety, myoclonus, seizures, anaphylaxis, arrhythmias, fatal anaphylaxis and anaphylactoid reactions, adrenal suppression and pain upon injection.
- Timing of AE Assessment: AEs will be assessed daily for 3 days (72 hours) after enrollment. In the event that a patient is discharged from the hospital before the 72-hour period is up, we will contact the patient by phone. Patients that remain in the hospital will be assessed for adverse events daily through review of the medical record and personal interview when possible. AEs will be reported to the Mayo Clinic IRB in a manner consistent with the site’s institutional policy.
- Annual AE and SAE Summaries: The study team will ensure that annual reports are submitted to the IRB and will contain (i) the number of adverse events and an explanation of how each event was handled, (ii) the number of complaints and how each complaint was handled, (iii) the number of withdrawals of study participants and an explanation for each withdrawal, and (iv) the number of protocol violations and how each was handled. Summaries of SAEs will be provided to the Data and Safety Monitoring Board (DSMB) at regular intervals, and DSMB reports and communications will be passed onto the Mayo Clinic Institutional Review Board.
- Safety Oversight: Safety oversight will be under the direction of a DSMB whose members will be independent from the study operations, and who will regularly review safety data throughout the study duration. Full details of the composition and the operation of the DSMB and how the safety analyses are to be performed will be detailed in a separate DSMB written charter. Enrollment may not begin, even with IRB and FDA approval, until the DSMB has reviewed and approved the protocol.
- Assessment of Toxicity: The study team will adopt the grading system toxicity as published by the FDA in September 2007 [44]. This severity scale will apply to any laboratory values or clinical abnormalities that fall outside of the institutions normal value range. The study investigator will sign off on the level of toxicity for any abnormal lab value or clinical symptom/sign.
- Assessment of Causality: The study team will adopt the WHO-UMC system for standardized case causality assessment [45].
- Adverse Event Coding: All adverse events will be coded using the MedDRA Database.
- Study Stopping Rules:
  - Futility: In order to ensure adequate statistical power for the primary analysis and not jeopardize the analyses of secondary outcomes, no interim analyses or early-stopping rules are included.
  - Safety: Judgment concerning the continuation or termination of the study will only be based on recommendations from the DSMB. The DSMB will play a valuable role in advising the study leadership on the relevance of advances in the diagnosis and treatment of patients. A number of therapeutic or diagnostic testing advances may possibly occur during the course of the trial. The DSMB will need to help put these advances in proper perspective. If protocol modifications are warranted, close consultation among the DSMB, the FDA, and the study Primary Investigator will be required. A separate DSMB charter that outlines in detail the operating guidelines for the committee and the protocol for evaluation of data will be developed prior to the start of patient randomization and agreed upon in the initial meeting of the DSMB. Minutes of all DSMB meetings will be prepared and promptly distributed to committee members and study sites. The principal investigator has the power to stop the study at any time.
- Immediate Study Subject Stopping Rules: The study will not delay intubation in patients. Clinicians will proceed per standard of care. In the event the study drug is not available by the time of intubation, the procedure will proceed in the usual fashion and the study drug will be wasted per standard policy and procedure. The study will effectively be stopped in such cases. In addition, if patients exhibit hemodynamic instability prior to study drug administration, the study will be stopped. Hemodynamic instability will be defined as (i) heart rate greater than 160 or less than 50 beats per minute; (ii) systolic blood pressure greater than 160 or less than 70 mmHg; and (iii) diastolic blood pressure greater than 100 or less than 30 mmHg.

The current study is under an IND through the FDA to satisfy the requirements for emergent use research. Because of FDA regulation, we were instructed to put forth safety thresholds or early stopping rules within the protocol. Truthfully, patients that exhibit the above criteria likely receive very little sedation and to sedate them may place them at risk.

## Discussion

The KEEP PACE trial will be the first randomized controlled trial to evaluate a ketamine and propofol combination in a 1:1 ratio against etomidate for urgent or emergent ETIs in critically ill patients. The trial will also be unique in that it will proceed with a waiver of informed consent due to the planned emergency use provision of the FDA for INDs. The primary outcome of interest will be hemodynamic stability in both groups. The trial will address a crucial gap in the literature regarding the optimal induction agent for ETI in patients that may have potential or established hemodynamic instability. The expected completion date is December 2015. Greater experience with planned emergency research will, hopefully, pave the way for future prospective randomized trials in the critically ill patient population. Our hope by publishing the current study protocol is to provide researchers who are considering planned emergent use research with tools to accomplish such a trial that adheres to both regulatory and institutional policies.

## Trial status

The trial is in the recruiting phase at the time of manuscript submission.

## Additional files

Additional file 1:
**Notices provided to patients and families in the hospital where the study is recruiting and advertisements placed in the local newspaper to the broader public.**
Additional file 2:
**Summary of trial overview provided to the clinical teams in the respective critical care units where the trial is recruiting.**
Additional file 3:
**Dose guidelines used within the current study.**
Additional file 4:
**Hemodynamic data collection and intubation difficulty data collection forms.**

## Abbreviations

AE: adverse event
ANOVA: analysis of variance
APACHE: Acute Physiology and Chronic Health Evaluation
CAM-ICU: Confusion Assessment Method for The Intensive Care Unit
CCaTS: Center for Clinical and Translational Science, Mayo Clinic, Rochester, MN
CRU: Clinical Research Unit, Mayo Clinic, Rochester, MN
DSMB: data and safety monitoring board
ETI: endotracheal intubation
FDA: United States Food and Drug Administration
ICU: intensive care unit
IDE: investigational device exemption
IND: investigational new drug
IRB: institutional review board
KEEP PACE: Ketamine/Propofol Admixture “Ketofol” at Induction in the Critically Ill Against Etomidate
MAP: mean arterial pressure
SAE: serious adverse event
