# Supplementary material for: Ketamine/propofol admixture (ketofol) at induction in the critically ill against etomidate (KEEP PACE trial): study protocol for a randomized controlled trial
Source: Trials. 2015 Apr 21;16:177. doi: 10.1186/s13063-015-0687-0 (PMC4409710; doi:10.1186/s13063-015-0687-0)
Supplement: Additional file 1: — Notices provided to patients and families in the hospital where the study is recruiting and advertisements placed in the local newspaper to the broader public. [file 13063_2015_687_MOESM1_ESM.docx]

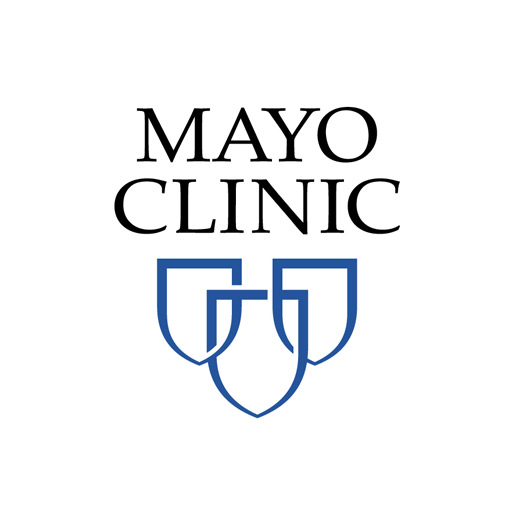


Turn to channel 236 in any of the 6 B/G patient or waiting rooms or visit <http://bit.ly/KEEPPACE> to find out more about an ongoing clinical trial that involves patients who are receiving emergency placement of a breathing tube.


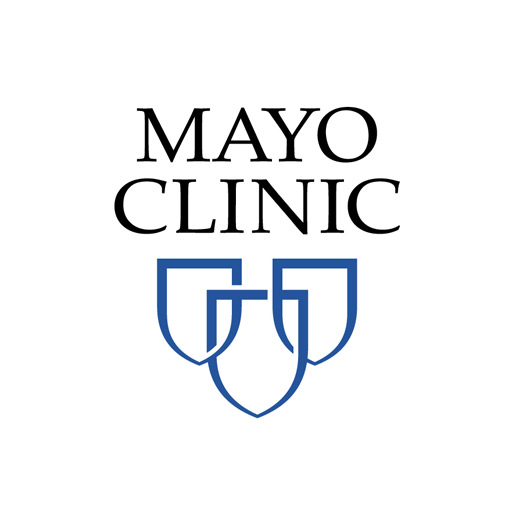


Turn to channel 236 in any of the 7 D/E patient or waiting rooms or visit <http://bit.ly/KEEPPACE> to find out more about an ongoing clinical trial that involves patients who are receiving emergency placement of a breathing tube.

Radio Ad (30 Second)

Mayo Clinic strives to be on the cutting edge of medical research in order to ensure that we are providing the best patient care possible. I am Dr. Nathan Smischney, and I want to let you know how you can learn more about a unique study that is currently taking place in the medical and surgical intensive care units at Mayo Clinic Hospital – Rochester, Saint Marys Campus. The goal of the study is to compare two types of anesthetic medications currently being used to facilitate the placement of a breathing tube which is a lifesaving procedure commonly needed during critical illness. Patients that may be included in the study are

- - Patients who are admitted in the Medical and Surgical Intensive Care Unit at Mayo Clinic Hospital – Rochester, Saint Marys Campus.

And

- - Require a breathing tube in an emergency situation.

To learn more, visit <http://bit.ly/KEEPPACE> or call 1-800-265-9263.

Newspaper Ad


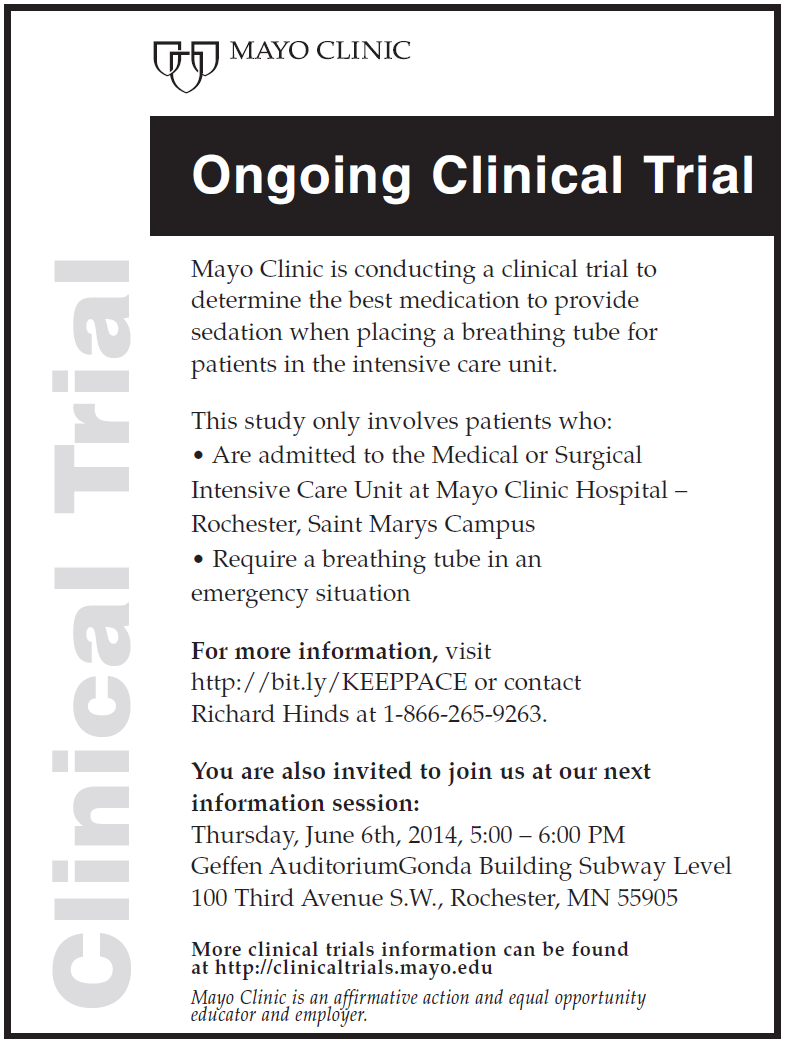
13433 Mayo Clinic Clinical Trial

3.75" w x 5" h = (2 col x 5" h)

– POST BULLETIN
