# Supplementary figures and images for: Ketamine/propofol admixture (ketofol) at induction in the critically ill against etomidate (KEEP PACE trial): study protocol for a randomized controlled trial
Source: Trials. 2015 Apr 21;16:177. doi: 10.1186/s13063-015-0687-0 (PMC4409710; doi:10.1186/s13063-015-0687-0)

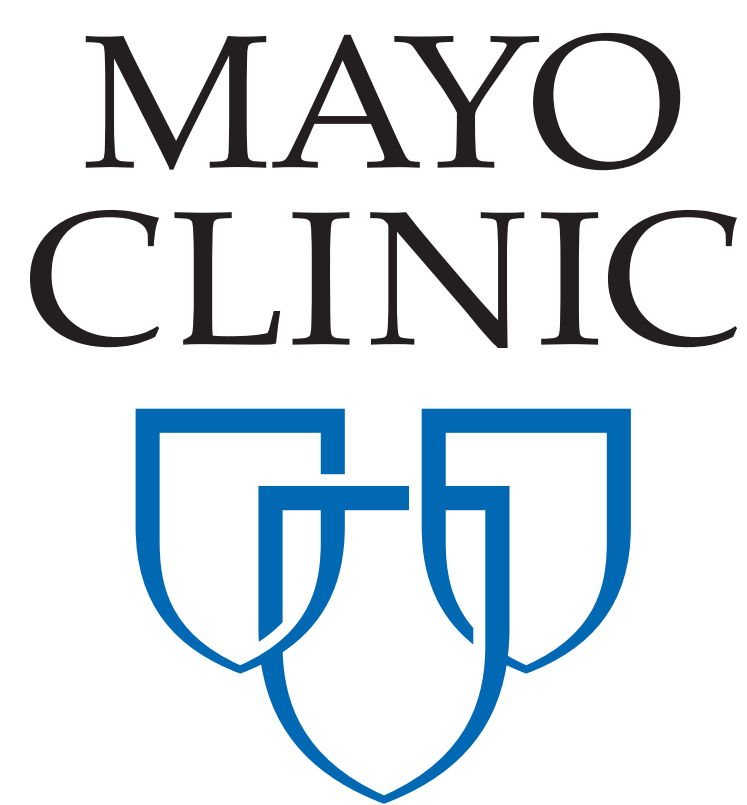

# KEEP PACE Trial

*Ketamine + Propofol vs. Etomidate for intubations in the ICU*

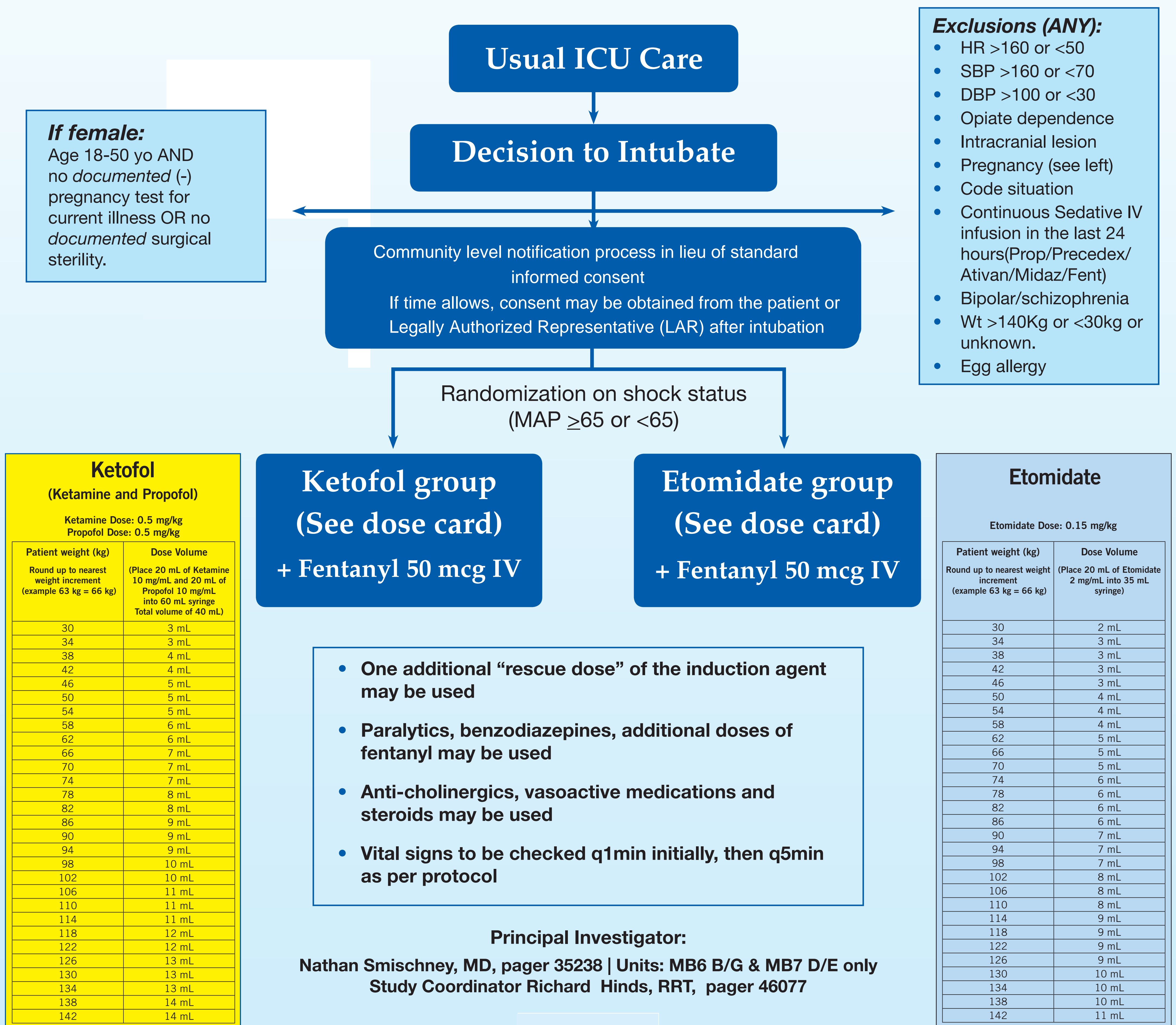

Supplement: Additional file 2: — Summary of trial overview provided to the clinical teams in the respective critical care units where the trial is recruiting. [file 13063_2015_687_MOESM2_ESM.pdf]
