# Supplementary material for: Ketamine/propofol admixture (ketofol) at induction in the critically ill against etomidate (KEEP PACE trial): study protocol for a randomized controlled trial
Source: Trials. 2015 Apr 21;16:177. doi: 10.1186/s13063-015-0687-0 (PMC4409710; doi:10.1186/s13063-015-0687-0)
Supplement: Additional file 3: — Dose guidelines used within the current study. [file 13063_2015_687_MOESM3_ESM.pdf]

KEEP PACE Dose Chart
IRB # 13-000506

Etomidate			Ketofol (Ketamine and Propofol)			
Etomidate Dose: 0.15 mg/kg			Ketamine Dose: 0.5 mg/kg<br>Propofol Dose: 0.5 mg/kg			
**Patient weight (kg)** Round up to nearest weight increment (example 63 kg = 66 kg)	**Dose Volume** (Place 20 mL of Etomidate 2 mg/mL into 35 mL syringe)	**Dose (mg)**	**Patient weight (kg)** Round up to nearest weight increment (example 63 kg = 66 kg)	**Dose Volume** (Place 20 mL of Ketamine 10 mg/mL and 20 mL of Propofol 10 mg/mL into 60 mL syringe Total volume of 40 mL)	**Dose (mg)**	
					**Ketamine**	**Propofol**
30	2 mL	4 mg	30	3 mL	15 mg	15 mg
34	3 mL	6 mg	34	3 mL	15 mg	15 mg
38	3 mL	6 mg	38	4 mL	20 mg	20 mg
42	3 mL	6 mg	42	4 mL	20 mg	20 mg
46	3 mL	6 mg	46	5 mL	25 mg	25 mg
50	4 mL	8 mg	50	5 mL	25 mg	25 mg
54	4 mL	8 mg	54	5 mL	25 mg	25 mg
58	4 mL	8 mg	58	6 mL	30 mg	30 mg
62	5 mL	10 mg	62	6 mL	30 mg	30 mg
66	5 mL	10 mg	66	7 mL	35 mg	35 mg
70	5 mL	10 mg	70	7 mL	35 mg	35 mg
74	6 mL	12 mg	74	7 mL	35 mg	35 mg
78	6 mL	12 mg	78	8 mL	40 mg	40 mg
82	6 mL	12 mg	82	8 mL	40 mg	40 mg
86	6 mL	12 mg	86	9 mL	45 mg	45 mg
90	7 mL	14 mg	90	9 mL	45 mg	45 mg
94	7 mL	14 mg	94	9 mL	45 mg	45 mg
98	7 mL	14 mg	98	10 mL	50 mg	50 mg
102	8 mL	16 mg	102	10 mL	50 mg	50 mg
106	8 mL	16 mg	106	11 mL	55 mg	55 mg
110	8 mL	16 mg	110	11 mL	55 mg	55 mg
114	9 mL	18 mg	114	11 mL	55 mg	55 mg
118	9 mL	18 mg	118	12 mL	60 mg	60 mg
122	9 mL	18 mg	122	12 mL	60 mg	60 mg
126	9 mL	18 mg	126	13 mL	65 mg	65 mg
130	10 mL	20 mg	130	13 mL	65 mg	65 mg
134	10 mL	20 mg	134	13 mL	65 mg	65 mg
138	10 mL	20 mg	138	14 mL	70 mg	70 mg
142	11 mL	22 mg	142	14 mL	70 mg	70 mg
