# Supplementary material for: Ketamine/propofol admixture (ketofol) at induction in the critically ill against etomidate (KEEP PACE trial): study protocol for a randomized controlled trial
Source: Trials. 2015 Apr 21;16:177. doi: 10.1186/s13063-015-0687-0 (PMC4409710; doi:10.1186/s13063-015-0687-0)
Supplement: Additional file 4: — Hemodynamic data collection and intubation difficulty data collection forms. [file 13063_2015_687_MOESM4_ESM.docx]

**KEEP PACE Trial**

**Data Collection Form**

*To be filled out by the lead respiratory therapist*


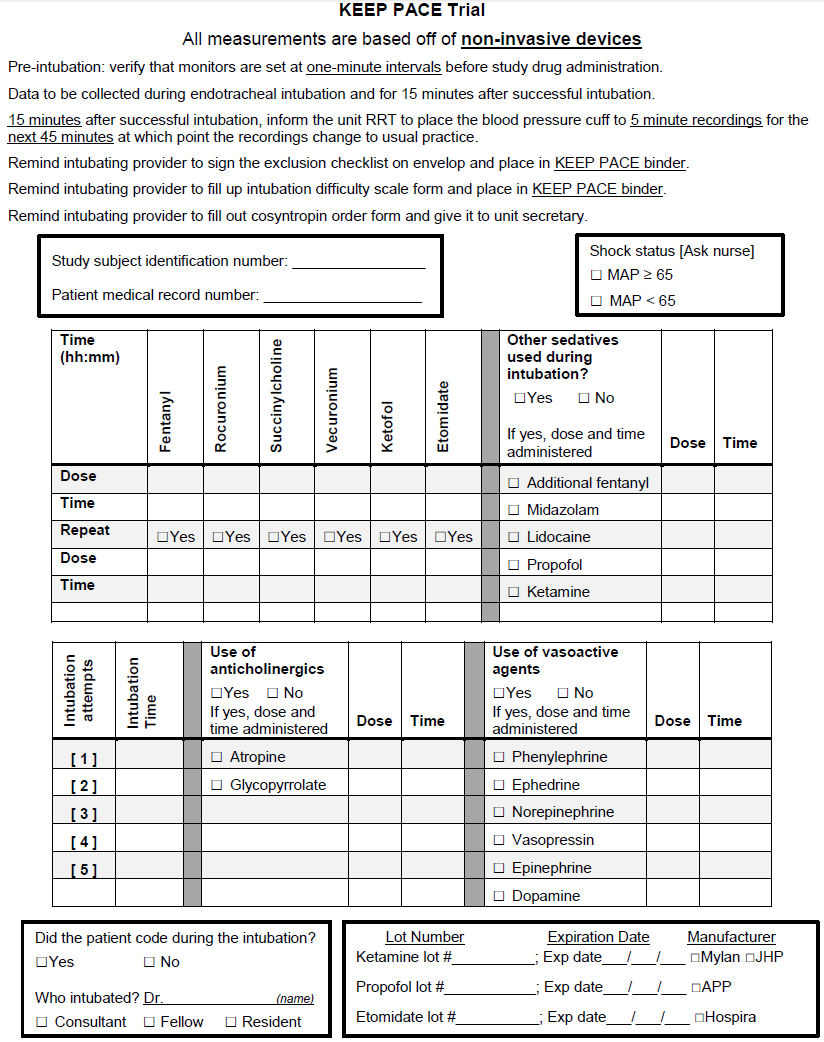


**KEEP PACE Trial**

**Intubation Difficulty Scale**

*To be filled out by the fellow/resident performing the intubation*

1. Number of intubation attempts: ___
2. Number of operators: ___
3. Number of alternative intubation techniques used: ___
4. Glottic exposure (Cormack and Lehane grade): ___


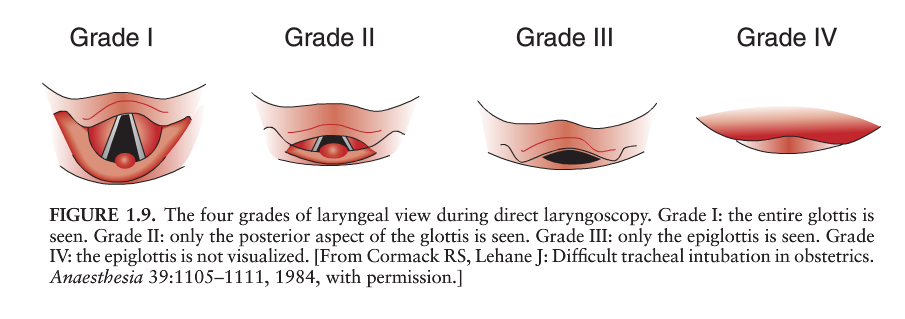


*Four grades of laryngeal view during direct laryngoscopy. Grade I: the entire glottis is seen. Grade II: only the posterior aspect of the glottis is seen. Grade III: only the epiglottis is seen. Grade IV: the epiglottis is not visualized.*

1. Lifting force required during laryngoscopy (0 = normal; 1 = increased) ___
2. Necessity for external laryngeal pressure (0 = not applied; 1 = applied) ___
3. Position of vocal cords at intubation (0 = Abduction/not observed; 1 = Adduction): ___
4. Reason for intubation (0=Airway protection; 1=Acute respiratory failure - dyspnea and/or Sa02 <90%; 2=Neurologic - stroke or altered mental status; 3=Shock - cardiac arrest; 4=other): ___
5. Were any of the following procedures performed (started or ongoing) **60 minutes** prior to the intubation? Are any of the following procedures planned within **60 minutes** following the intubation? [mark ‘X’ in the appropriate boxes]

| **Procedure** | **60 min PRE-Intubation** | | | **60 min POST-Intubation** | | |
| --- | --- | --- | --- | --- | --- | --- |
|  | **Yes** | **No** | **Unsure** | **Yes** | **No** | **Unsure** |
| Central line placement (including PICC line) |  |  |  |  |  |  |
| GI Endoscopy (EGD, colonoscopy, sigmoidoscopy, ERCP etc.) |  |  |  |  |  |  |
| Bronchoscopy |  |  |  |  |  |  |
| Any other procedure |  |  |  |  |  |  |
| Other – please specify |  | | |  | | |

**References:**

- McElwain J, Simpkin A, Newell J, Laffey JG. Determination of the utility of the Intubation Difficulty Scale for use with indirect laryngoscopes. *Anaesthesia*. 2011; 66:1127-1133.
- Cormack RS, Lehane J. Difficult tracheal intubation in obstetrics. *Anaesthesia*. 1984; 39:1105-1111.
- Adnet F, Borron SW, Racine SX, Clemessy JL, Fournier JL, Piaisance P, Lapandry C. The intubation difficulty scale (IDS): proposal and evaluation of a new score characterizing the complexity of endotracheal intubation. *Anesthesiology*. 1997; 87:1290-1297.
